# Comment on “binge drinking and alcohol prices”

**DOI:** 10.1186/s13561-016-0082-x

**Published:** 2016-01-26

**Authors:** Ziming Xuan, Thomas F. Babor, Timothy S. Naimi, Jason G. Blanchette, Frank J. Chaloupka

**Affiliations:** 1 Boston University School of Public Health, Boston, MA USA; 2 University of Connecticut School of Medicine, Farmington, CT USA; 3 Boston Medical Center, Boston, MA USA; 4 University of Illinois at Chicago, Chicago, IL USA; 5 Department of Community Health Sciences, Boston University School of Public Health, 801 Massachusetts Avenue, Boston, MA 02118 USA

## To the editors of health economics review:

There is ample evidence on the effects of prices and taxes on heavy drinking, including binge drinking [1]. Experiments on “Happy Hour” discounts in barroom settings have shown that when the price of alcohol decreases, consumption increases, and vice versa [2, 3]. A well-cited meta-analysis of 112 studies (Wagenaar et al., [4]) identified ten studies on heavy drinking and estimated a significant elasticity of −0.28. Another systematic review by Elder and colleagues [5] concluded that alcohol tax levels were inversely associated with excessive drinking. This is consistent with the conclusion from a widely-cited review by Cook and Moore [6] that “an increase in price results in reduced consumption”, and this applies to drinking by youth, heavy drinkers, and alcoholics who develop cirrhosis due to chronic consumption. Xuan and colleagues [7] showed that an improved comprehensive measure of alcohol taxes including specific excise tax and value-based taxes resulted in more negative tax elasticity and price elasticity predicting binge drinking, as compared to a conventional measure that relies only on beer excise tax. Another meta-analysis by Wagenaar and colleagues [8] showed that increased taxes and prices were associated with reduced alcohol-related disease and injury outcomes that are attributable to binge drinking.

Nevertheless, in an issue of Health Economics Review, Dr. Jon Nelson published a “systematic” review article with the following conclusion: “Increased alcohol taxes or prices are unlikely to be effective as a means to reduce binge drinking, regardless of gender or age group.” [9]. We have reviewed this paper based on its methodology, study interpretation, and conclusion.

### Faulty methodology

There is a general consensus that the scientifically recommended approach to estimate an overall effect across multiple studies is meta-analysis. Meta-analysis is the final step in the standardized protocol detailed in Preferred Reporting Items for Systematic Reviews and Meta-Analyses (PRISMA), which was cited in the Nelson paper. However, instead of conducting a rigorous meta-analysis among the 56 studies that he manually selected and screened, Dr. Nelson deviated from the PRISMA approach by simply counting the number of studies with p ≤ 0.05. Dr. Nelson’s approach failed to account for the heterogeneity of the effect sizes and sample variation due to different studies, and potential publication bias. Dr. Nelson stated that due to “diversity of models and results,” quantitative coefficient estimates for a meta-analysis were not collected. Dr. Nelson’s reference to “diversity of models and results” is the exact reason why quantitative meta-analysis is necessary to obtain a common effect estimate in the first place.

Furthermore, among the exclusion criteria, Dr. Nelson stated that studies were excluded if they are “based on a laboratory experiment” or use an interrupted time-series analysis. He did not explain why these two research designs were excluded. Not only do they have better internal validity than other designs, interrupted time series analysis is an ideal method for evaluating natural experiments, a type of study included in Dr. Nelson’s paper and stated in the article title.

### Misconstrued interpretations

Based on his characterization of our own work, there is considerable evidence of misconstrued presentation of the literature in Dr. Nelson’s article. For example, Dr. Nelson cited a study by Xuan et al. [10] and stated “Not signif. w/ adult binge incl.” in Table 2 in Dr. Nelson’s article. This representation of the study finding is incorrect. Xuan et al. [10] found that a higher beer tax was associated with significantly lower odds of youth binge drinking among the participants of biennial Youth Risk Behavior Surveys from 1999 to 2009, and the effect of alcohol taxes on reduced youth binge drinking was mediated (explained) in part through its effect on reducing adult binge drinking. In other words, adult binge drinking was being assessed as a possible mediator on the causal pathway between alcohol tax and youth binge drinking, and was not a covariate. Journal readers, based on Dr. Nelson’s statement of non-significance, were therefore erroneously informed that this study did not find a significant inverse relationship between tax and youth binge drinking when the opposite was the case.

Dr. Nelson further asserted that evidence in several widely-cited studies is drawn from aggregate econometric studies, and here he cited another one of our studies (Nelson TF et al., [11]). His assertion was incorrect because this study was based on ratings of the efficacy of various alcohol policies. This study was clearly not an econometric study per se, nor were the policy efficacy rating data drawn from econometric studies.

### Inadequate literature review

Dr. Nelson subsequently stated that “price and tax elasticity estimates for general populations may not apply equally to binge drinkers and other excessive drinkers.” To support this statement, Dr. Nelson cited Ayyagari and colleagues [12], which was a study restricted to individuals 50 years of age and older. Given the fact that binge drinking prevalence is highest among young adults, the paper cited by Dr. Nelson offers little support for his claim. He further cited a book chapter by Cook and Moore [13] without considering later work by Cook and Moore [6], as cited in the introduction of this letter. Equal effects are not necessary for establishing the critical importance of taxes in reducing excessive consumption and related harms. A more recent study showed that higher alcohol taxes including specific excise tax and value-based taxes were significantly associated with reduced binge drinking among the adult population in United States [7].

### Conflict of interest

Dr. Nelson disclosed that “Research leading to this paper was supported in part by the International Center for Alcohol Policies, Washington, DC.” However, the author failed to disclose his role as a consultant (2012–2015) and member of the Research Advisory Board of ICAP, an alcohol industry-funded “social aspect organization”. ICAP-sponsored research has been criticized for faulty methodology and erroneous interpretations [14]. Nor does Dr. Nelson declare that he has received consulting fees from several law firms that represent companies in the alcohol industry on issues related to this paper.

### Summary

In summary, Dr. Nelson’s conclusion that taxes were unlikely to be effective at reducing binge drinking is based on faulty methodology and an incorrect interpretation of the findings from studies, and his conclusion contradicts the extensive scientific literature. Binge drinking causes more than half of nearly 80,000 alcohol-attributable deaths in the U.S. each year and accounts for three-quarters of the 224 billion dollars in annual economic costs [15]. This is not a trivial public health issue. Alcohol taxation has been one of the few policy levers that can have substantial benefits on population health. To cast doubt on a near-consensus in the alcohol literature using faulty reasoning and inappropriate review methods does a disservice to science as well as public health.
